# Supplementary figures and images for: NLRP3 and AIM2 inflammasomes exacerbate the pathogenic Th17 cell response to eggs of the helminth Schistosoma mansoni
Source: PLoS Pathog. 2025 Mar 18;21(3):e1012108. doi: 10.1371/journal.ppat.1012108 (PMC11918320; doi:10.1371/journal.ppat.1012108)

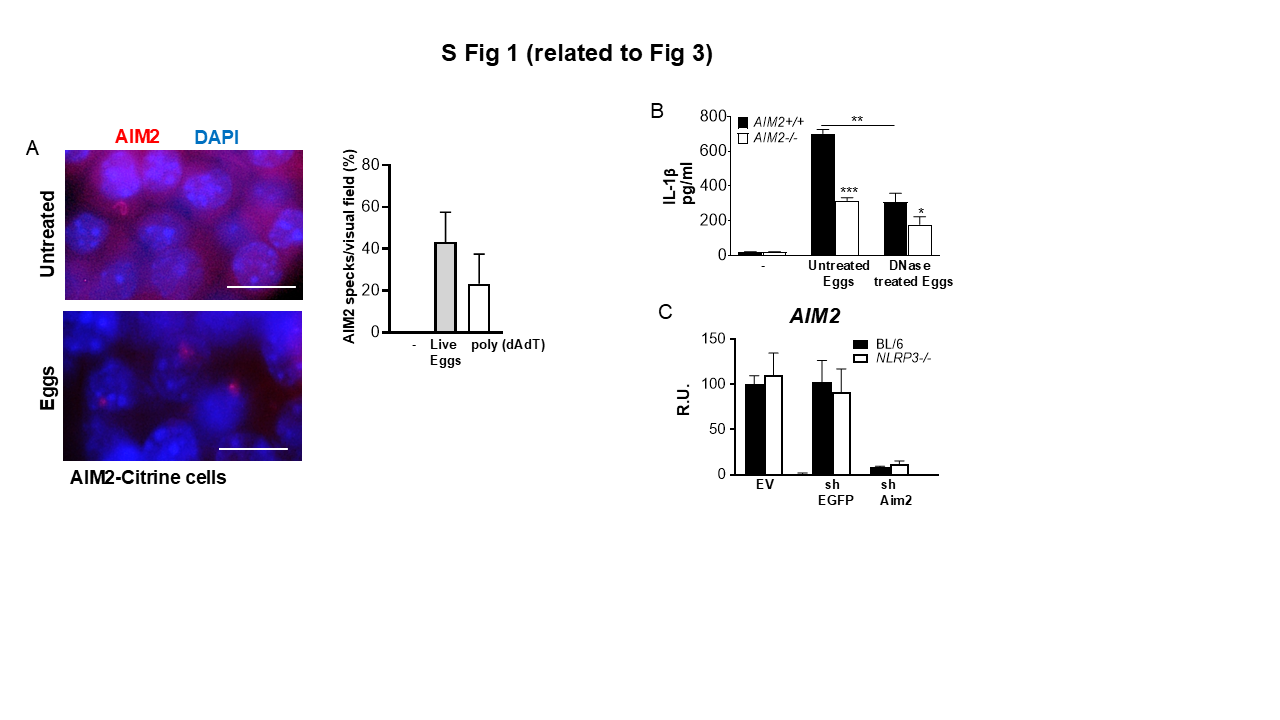

Supplement: S1 Fig — Scale bar: 20μm (top), 20μm (bottom). DAPI was used to stain the nuclei of the cells. The formation of AIM2 pyroptosomes was quantified (right). Data are representative of at least 15 fields of view and three independent experiments. (B) Stimulation of BMDCs with DNase-treated eggs leads to reduced IL-1β production. BMDCs from Aim2+/+ and Aim2-/- mice were cultured for 24h with 100 live eggs or DNase-treated eggs. IL-1β in supernatants was measured by ELISA. Bars represent the mean +/- SD cytokine levels of three biological replicates from one representative experiment of two with similar results. *p <0.05, **p <0.005, ***p <0.0005. (C) Verification of AIM2 knockdown in BMDCs. BL/6 and NLRP3-/- BMDCs were transduced with empty vector (EV), EGFP shRNA (shEGFP) or AIM2 shRNA (shAIM2). The mRNA levels of AIM2 were set at 100% in cells transduced with EV. AIM2 mRNA levels were assessed by qRT-PCR. Bars represent the mean ±S.D. AIM2 relative units (R.U.) of three biological replicates from one representative experiment of three with similar results. (TIF) [file ppat.1012108.s001.tif]

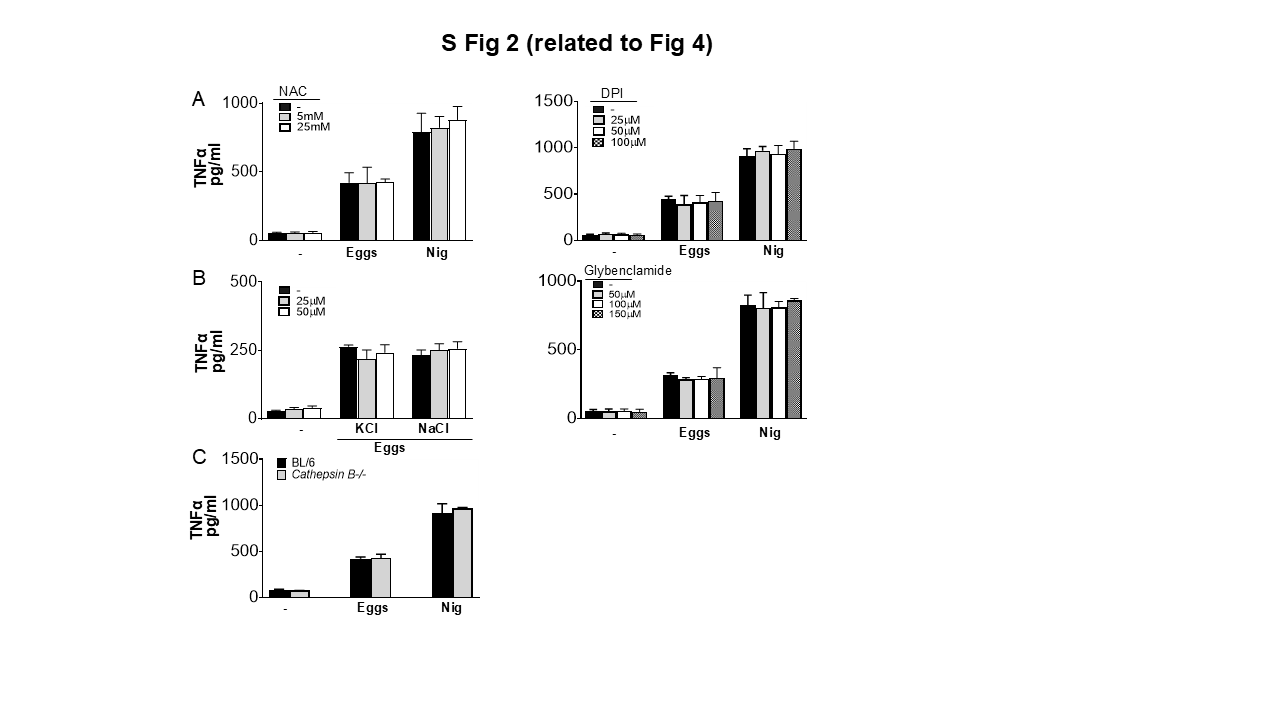

Supplement: S2 Fig — (A) BL/6 BMDCs were pretreated with the indicated concentrations of ROS inhibitor N-acetyl-l-cysteine (NAC) (left) or Diphenyleneiodonium (DPI) (right) for 1h before culturing for 24h with 100 live (or no) eggs, or LPS plus Nig. TNFα in supernatants was measured by ELISA. (B) BL/6 BMDCs were pretreated with indicated concentrations of potassium chloride (KCl), sodium chloride (NaCl) (left) or potassium channel blocker Glybenclamide (right) for 1h before culturing for 24h with 100 live (or no) eggs, or LPS plus Nig. TNFα in supernatants was measured by ELISA (C) BMDCs from BL/6 and Cathepsin B-/- mice were cultured for 24h with 100 live (or no) eggs or LPS plus Nig. TNFα in supernatants was measured by ELISA. Bars represent the mean +/- SD cytokine levels of three biological replicates from one representative experiment of two with similar results. (TIF) [file ppat.1012108.s002.tif]

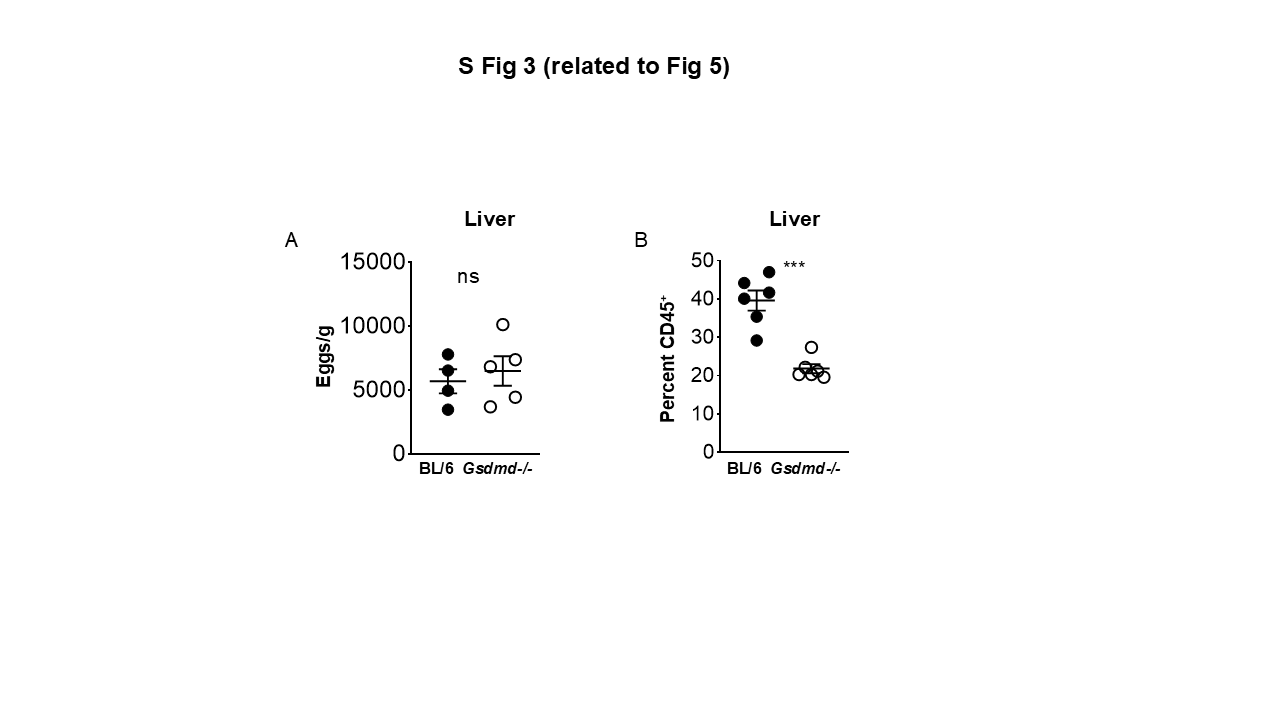

Supplement: S3 Fig — Number of eggs were microscopically counted in pieces of weighted liver from BL/6 and Gsdmd-/- mice (B) Total CD45+ populations were decreased in the Gsdmd-/- livers. Total CD45+ populations in liver cells isolated from C57BL/6 and Gsdmd-/- mice infected with Schistosoma mansoni for 7 weeks. Data are representative of two independent experiments. Significance was determined using a Student’s t-test ***p < 0.0005. (TIF) [file ppat.1012108.s003.tif]
